# Genetic Evolution during the development of an attenuated EIAV vaccine

**DOI:** 10.1186/s12977-016-0240-6

**Published:** 2016-02-03

**Authors:** Xue-Feng Wang, Yue-Zhi Lin, Qiang Li, Qiang Liu, Wei-Wei Zhao, Cheng Du, Jie Chen, Xiaojun Wang, Jian-Hua Zhou

**Affiliations:** State Key Laboratory of Veterinary Biotechnology, Harbin Veterinary Research Institute, Chinese Academy of Agricultural Sciences, Harbin, 150001 China; Department of Preventive Veterinary Medicine, College of Veterinary Medicine, Northeast Agricultural University, Harbin, China; Harbin Weike Biotechnology Development Company, Harbin, China; Harbin Pharmaceutical Group Biovaccine Co., Harbin, 150069 China

**Keywords:** EIAV, Genetic Evolution, Lentiviral vaccine

## Abstract

**Background:**

The equine infectious anemia virus (EIAV) vaccine is the only attenuated lentiviral vaccine applied on a large scale that has been shown to be effective in controlling the prevalence of EIA in China. This vaccine was developed by successive passaging of a field-isolated virulent strain in different hosts and cultivated cells. To explore the molecular basis for the phenotype alteration of this vaccine strain, we systematically analyzed its genomic evolution during vaccine development.

**Results:**

Sequence analysis revealed that the genetic distance between the wild-type strain and six representative strains isolated from key development stages gradually increased with the number of passages. *Env* gene, but not *gag* and *pol*, showed a clear evolutionary flow similar to that of the whole genomes of different generations during the attenuation. Stable mutations were identified in multiple regions of multiple genes along with virus passaging. The adaption of the virus to the growth environment of cultured cells with accumulated genomic and genetic variations was positively correlated with the reduction in pathogenicity and rise of immunogenicity. Statistical analyses revealed significant differences in the frequency of the most stable mutations between in vivo and ex vivo-adapted strains and between virulent and attenuated strains.

**Conclusions:**

These data indicate that EIAV evolution during vaccine development generated an accumulation of mutations under the selective drive force, which helps to better understand the molecular basis of lentivirus pathogenicity and immunogenicity.

**Electronic supplementary material:**

The online version of this article (doi:10.1186/s12977-016-0240-6) contains supplementary material, which is available to authorized users.

## Background

Vaccination is the most effective means of controlling infectious diseases. However, the development of a safe and effective lentiviral vaccine, such as a human immunodeficiency virus-1 (HIV-1) vaccine, remains a huge scientific challenge. Studies on the development of lentiviral vaccines targeting HIV-1, simian immunodeficiency virus (SIV), chimeric simian-human immunodeficiency virus (SHIV), equine infectious anemia virus (EIAV) and feline immunodeficiency virus (FIV) have demonstrated that the live attenuated formulation is the most effective formulation [1, 2]. Because lentiviral genomes integrate into host chromosomes and feature a high frequency of genomic mutations, attenuated live vaccines are generally not considered as an option for practical lentiviral vaccines. However, the study of immune responses induced by attenuated vaccines can provide a useful reference to elucidate the protective immune responses to lentiviral infections [3].

EIAV is a member of the *Lentivius* genus in the *Retroviridae* family. The major features of EIAV, including its genomic structure, life cycle, in vivo antigen evolution, cell tropism and the interaction between the virus and host, are similar to HIV-1. Most horses infected with EIAV exhibit a repeated high-titer viremia with typical clinical features such as fever, thrombocytopenia and anemia. Some infected horses may eventually control viral replication and become asymptomatic carriers after several months or years of acute or chronic infection. EIAV infection is life-long.

**Fig. 1:**
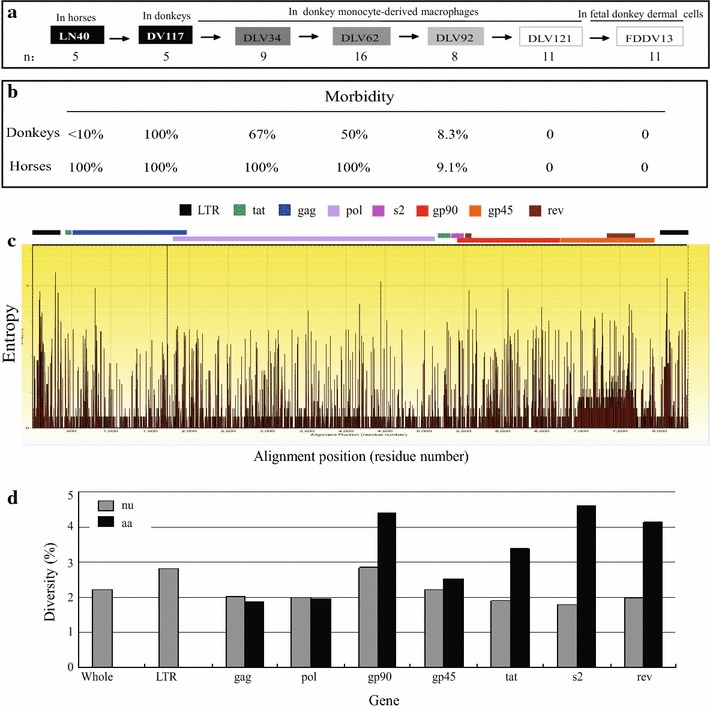
Genome variants of EIAV strains during vaccine development. **a** The flowchart of EIAV vaccine development. Four major stages were included the process of vaccine development: the in vivo passages in horses and donkeys and the passages in cultured dMDM and fetal donkey dermal (FDD) cells. The representative strains isolated from each of these stages and the numbers of clones used for the proviral genome analysis are indicated. These viral strains included the virulent strains EIAV_LN40_ and EIAV_DV117_, the vaccine strains EIAV_DLV121_ and EIAV_FDDV13_ and three strains of EIAV_DV117_ passaged in dMDM (the 34th passage strain EIAV_DLV34_, the 62nd passage strain EIAV_DLV62_ and the 92nd passage strain EIAV_DLV92_). **b** The pathogenicity attenuation of EIAV strains during vaccine development. The percentages indicate the morbidity of horses or donkeys that were experimentally infected with the indicated viral strains. **c** Shannon entropy (SE) plots of EIAV complete genomic sequences depict the mutation frequency at each nucleotide position. Full genomic sequences of 65 clones from seven EIAV strains were isolated from different key stages of vaccine development. Refer to Fig. 1a for detailed information of these viral strains. Larger entropy values indicate higher mutation frequencies. The relative positions of EIAV individual genes are labeled. **d** The variations in the EIAV genome and individual genes and their encoded proteins among the 65 clones from the seven representative strains. The diversities were calculated by computing the overall mean distances using the p-distance method in MEGA 5 software

EIAV_LN40_, which is an EIAV strain that is highly lethal to horses (experimental lethality of 80–100 %), was developed by passaging a field isolate in horses for 16 passages. The pathogenicity of this virus in donkeys was largely enhanced (raised from generally asymptomatic to near 100 % lethality) by the continuous passaging of EIAV_LN40_ in donkeys for 117 rounds (Fig. 1a). The resultant strain (EIAV_DV117_) was subsequently continuously acclimatized in cultivated primary donkey monocyte-derived macrophages (dMDM). The lethality of EIAV_DV117_ to horses and donkeys was gradually decreased by passaging in dMDM and was finally reduced to the point of not causing any clinical symptoms in either horses or donkeys after 90 passages (Fig. 1b). The ability to induce protective immunity in inoculated horses was detected in viruses that were attenuated in dMDM for approximately 110–125 passages. The resultant viral strain (termed EIAV_DLV121_) was able to elicit resistance to challenge with EIAV_LN40_ (the average genomic divergence to EIAV_DLV121_ was 2.8 %) in >75 % of the inoculated horses and >95 % of the inoculated donkeys. This attenuated viral strain was used as a vaccine to massively immunize 61 million horses and mules to prevent EIA infection in China from 1975 to the 1990s [4]. This nationwide vaccination program ended the incidence of equine infectious anemia (EIA) in this country. The successful application of the EIAV vaccine has provided an important and unique reference model for studies on lentivirus immunity and vaccines. EIAV_DLV121_ was further adapted to cultivated fetal donkey dermal (FDD) cells (termed EIAV_FDDV13_) to reduce the preparation costs of this attenuated EIAV strain. Part of the historic data on EIAV_DLV121_ protection of disease in laboratory infected horses indicated that the protection efficiency to challenge with the parental virulent strain (EIAV_LN40_, the average variation in Env amino acid sequences is 7.1 %) and an American strain (EIAV_Wyoming_, the average variation in Env amino acid sequences is 37.8 %) was 81 % (25/31) and 80 % (8/10), respectively [4]. Our resent experiments on the immunogenicity of EIAV_DLV121_ and EIAV_FDDV13_ demonstrated a 50 % (2/4) and 83 % (5/6) similar protection of disease (Additional file 1: Table S1) [4, 5].

During the development process of the attenuated EIAV vaccine, a series of virus strains with different pathogenicities or immunogenicities were obtained. These strains provided a useful resource for the study of essential factors that induce protective immunity to lentiviruses. In this article, we analyzed the proviral genomic characteristics and the evolutionary trend of representative strains from key stages of the process, including EIAV_LN40_, EIAV_DV117_, EIAV_DLV34_, EIAV_DLV62_, EIAV_DLV92_, EIAV_DLV121_ and EIAV_FDDV13_. Among these strains, EIAV_DLV34_, EIAV_DLV62_ and EIAV_DLV92_ were collected from the 34th, 62nd and 92nd passages of EIAV_DV117_ in dMDM, respectively (Fig. 1a).

## Results and discussion

### Analysis of viral genome variants over the course of the development of an attenuated EIAV vaccine

To examine the overall contributions of genes or gene fragments of the EIAV genome to the evolution of this virus during the development of the vaccine strains EIAV_DLV121_ and EIAV_FDDV13_, the frequency of each nucleotide of 65 full genomic sequences of EIAV strains sampled from key stages of the process was analyzed using Shannon Entropy (SE). As presented in Fig. 1c, nucleotides with high SE values were not randomly distributed but were clustered as different-sized islands. These nucleotides were largely located in the LTR and *env* regions.

The proviral genome size of different EIAV strains ranged from 7549 to 8277 bp. A detailed analysis of the average mutation rates of different genes and the LTR revealed that the overall diversity among the total 65 genomes of the seven EIAV strains was 2.0 %. The diversity was highest in the LTR (2.95 ± 0.26 %), followed by *env* (consisting of the *gp90* and *gp45* genes, which were 2.90 ± 0.19 and 2.11 ± 0.26 %, respectively) (Fig. 1d). The variation in the encoded proteins among these EIAV strains was considerably higher. The S2 accessary protein exhibited the highest diversity in the amino acid sequence, reaching 5.02 ± 1.58 %, followed by the surface unit (SU or gp90) of the envelope protein (Env) and Rev, which were 4.81 ± 0.48 and 3.99 ± 0.84 %, respectively (Fig. 1d). Moreover, the genetic distances between the different viral strains and the parental strains EIAV_LN40_ and EIAV_DV117_ gradually increased with the increasing passage numbers (Table 1).

**Table 1:** Comparison of nucleotide and amino acid genetic distances of the various EIAVs compared to EIAV_LN40_ and EIAV_DV117_

		EIAV_LN40_						EIAV_DV117_				
		EIAV_DV117_	EIAV_DLV34_	EIAV_DLV62_	EIAV_DLV92_	EIAV_DLV121_	EIAV_FDDV13_	EIAV_DLV34_	EIAV_DLV62_	EIAV_DLV92_	EIAV_DLV121_	EIAV_FDDV13_
LTR	nu	2.90 (0.85)^a^	3.30 (0.84)	4.18 (1.11)	4.18 (1.16)	4.06 (1.01)	5.86 (1.40)	1.94 (0.58)	2.97 (0.90)	3.68 (1.01)	2.59 (0.77)	3.71 (1.09)
Gag	nu	2.02 (0.35)	1.72 (0.26)	1.96 (0.29)	2.16 (0.31)	2.30 (0.32)	2.47 (0.37)	2.13 (0.29)	2.31 (0.31)	2.58 (0.34)	2.62 (0.32)	2.57 (0.36)
	aa	1.91 (0.51)	1.69 (0.42)	2.09 (0.53)	2.08 (0.53)	2.73 ± (0.58)	2.31 (0.57)	1.98 (0.45)	2.12 (0.50)	2.25 (0.53)	2.73 (0.54)	2.14 (0.50)
Pol	nu	1.99 (0.24)	1.93 (0.16)	2.00 (0.10)	2.06 (0.19)	2.40 (0.20)	2.75 (0.23)	1.65 (0.16)	1.77 (0.16)	1.74 (0.17)	2.13 (0.19)	2.38 (0.23)
	aa	2.03 (0.41)	2.13 (0.34)	2.27 (0.34)	2.38 (0.36)	2.49 (0.38)	2.84 (0.43)	1.35 (0.23)	1.69 (0.26)	1.61 (0.26)	1.79 (0.27)	2.00 (0.32)
gp90	nu	3.13 (0.43)	3.28 (0.39)	3.40 (0.41)	3.51 (0.41)	3.94 (0.44)	4.01 (0.48)	3.43 (0.40)	3.46 (0.42)	3.60 (0.43)	3.99 (0.43)	3.78 (0.46)
	aa	4.20 (0.91)	5.17 (0.82)	5.17 (0.87)	5.55 (0.91)	6.07 (0.90)	6.71 (1.11)	6.38 (0.93)	6.20 (0.94)	6.38 (0.97)	7.05 (1.04)	7.59 (1.18)
gp45	nu	2.45 (0.38)	2.28 (0.29)	2.36 (0.26)	2.40 (0.29)	2.60 (0.31)	2.86 (0.41)	1.64 (0.23)	1.89 (0.23)	1.86 (0.27)	2.18 (0.29)	2.32 (0.40)
	aa	3.31 (0.69)	3.14 (0.55)	3.56 (0.50)	3.22 (0.53)	3.42 (0.53)	–^b^	1.78 (0.41)	2.60 (0.44)	2.14 (0.47)	2.71 (0.53)	–
Tat	nu	1.62 (0.73)	1.13 (0.36)	1.40 (0.45)	1.74 (0.57)	2.04 (0.67)	2.27 (0.79)	2.28 (0.72)	2.44 (0.75)	2.59 (0.78)	2.61 (0.80)	2.85 (0.90)
	aa	2.05 (1.26)	2.26 (0.97)	2.93 (1.27)	3.76 (1.51)	4.38 (1.91)	5.11 (2.11)	2.98 (1.36)	3.29 (1.45)	3.81 (1.53)	3.19 (1.61)	3.93 (1.83)
S2	nu	1.97 (0.91)	1.72 (0.48)	2.14 (0.77)	2.45 (0.88)	2.94 (1.01)	2.45 (0.98)	3.00 (0.96)	3.31 (1.11)	3.12 (1.07)	4.10 (1.29)	2.85 (1.10)
	aa	3.60 (2.20)	6.85 (2.51)	8.08 (3.23)	7.57 (2.99)	10.61 (3.81)	6.38 (3.10)	4.17 (1.36)	5.62 (2.47)	6.78 (2.75)	7.97 (3.09)	6.38 (3.13)
Rev	nu	1.62 (0.50)	1.61 (0.37)	1.81 (0.33)	1.50 (0.34)	1.81 (0.35)	2.23 (0.65)	1.57 (0.39)	1.94 (0.39)	1.59 (0.39)	1.86 (0.38)	1.58 (0.54)
	aa	2.52 (0.77)	3.29 (0.72)	2.48 (0.63)	3.64 (0.84)	5.70 (1.87)	3.47 (0.12)	4.25 (0.11)	3.66 (0.11)	4.45 (0.10)	4.77 (0.63)	2.52 (0.77)
Complete		2.29 (0.12)	2.22 (0.13)	2.37 (0.17)	2.56 (0.11)	2.78 (0.10)	3.15 (0.11)	2.10 (0.11)	2.25 (0.15)	2.38 (0.12)	2.57 (0.13)	2.81 (0.17)

^a^The number in parenthesis indicate standard deviation

^b^The gp45 amino acid genetic distances of EIAV_FDDV13_ with EIAV_LN40_ and EIAV_DV117_ no showed, because existence of truncated gp45

### Phylogenetic analysis of strains collected during the development of an attenuated EIAV vaccine

**Fig. 2:**
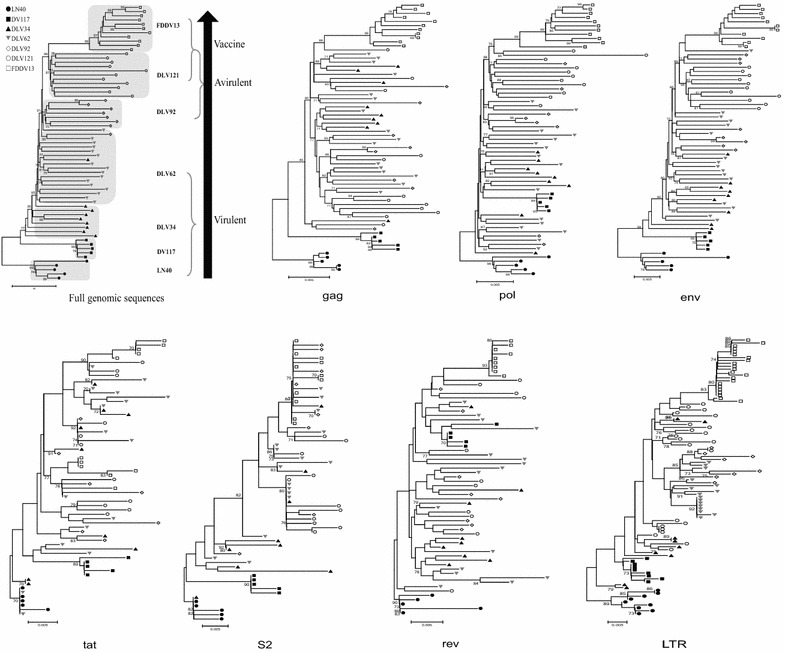
Phylogenetic tree analysis of EIAV proviral genomes and individual genes (*gag*, *pol*, *env,* *tat, s2, rev* and LTR). Sequence data were collected from 65 complete proviral genomes of seven viral strains. The phylogenetic trees were constructed using the neighbor-joining method and calculated with the Kimura 2 parameter in MEGA 5. Bootstrap values above 70 % are indicated. The virulent strains, including EIAV_LN40_, EIAV_DV117_, EIAV_DLV34_ and EIAV_DLV62_, are viruses that cause typical EIA symptoms (rectal temperature >39 °C, the platelet count <100,000/μL and the plasma viral load >10^6^ copies/mL)

Phylogenetic trees were constructed based on the 65 full-length proviral genomic sequences (Fig. 2). The clusters of each individual genome clearly demonstrated the evolutionary direction from EIAV_LN40_ to EIAV_FDDV13_. Changes in the viral growth environment inevitably created new branches in the phylogenetic tree [i.e., changing the host from horse to donkey (between EIAV_LN40_ and EIAV_DV117_) and shifting the cultivated cell type from dMDM to FDD (between EIAV_DLV121_ and EIAV_FDDV13_)]. Conversely, the evolution of EIAV was rather gradual and smooth under the same growth environment conditions, as shown by the overlap of the sequence distributions of species from EIAV_DLV34_, EIAV_DLV62_ and EIAV_DLV92_ in the map. This result indicated that viral evolution was a long-term process with selective mutations accumulating during continuous passages in cultured cells. Furthermore, dramatic alterations of the growth environment could speed up viral genomic mutations.

The evolutionary behavior of each individual EIAV gene and the LTR region were also phylogenetically analyzed. Three different evolutionary patterns were observed, as presented in Fig. 2. The first pattern was observed for the *env* gene and clearly demonstrated a direction toward the flow of the vaccine development process similar to that identified for the total viral genome, with a remarkable clustering of sequences from attenuated strains (EIAV_DLV92_, EIAV_DLV121_ and EIAV_FDDV13_) (Fig. 2). This result implied that there was a close association of the *env* evolution with the EIAV phenotypic change during the in vivo and ex vivo passages. The *pol* sequences also displayed a separated distribution between the EIAV virulent and attenuated strains (Fig. 2). However, the genes were not well-separated among strains with a similar pathogenicity, suggesting a close association with pathogenicity. The second pattern was observed for LTR and *gag* (Fig. 2), which showed separated branches of sequences that clustered according to the different EIAV growth environments. Specifically, sequences of EIAV_LN40_ (from horses), EIAV_DV117_ (from donkeys) and EIAV_FDDV13_ (from FDD cells) each formed a clearly separated branch. Although clones from strains grown in eMDM (EIAV_DLV_ strains 34, 62, 92 and 121) were well-distinguished from the other strains, sequences within these EIAV_DLV_ strains were mixed in the sub-branches of their clusters. This phylogenetic pattern suggested the association of LTR and *gag* with EIAV adaption to the environmental change and the reduced importance of pathogenicity. The third genetic evolutionary pattern was observed for the genes of three accessory proteins (*tat*, *s2* and *rev*) (Fig. 2). For *tat* and *rev* in particular, the sequence distribution in the phylogenetic tree did not follow the trend of in vivo and ex vivo passaging of EIAV. Viral genes in different passages were largely mixed within different branches, demonstrating no obvious evolutionary direction. However, *s2* exhibited a separation between sequences from strains replicated either in vivo or cultured cells (Fig. 2).

Combined with Fig. 1d, which depicts the higher amino acid mutation rate, our data on EIAV genetic evolution suggest that: (1) Gag, Pol and accessary proteins have higher percentages of silent nt substitutions or substitutions that are not consistent with the substitution patterns of the whole genome during the alteration of growth environment, implying that they are less driven by selective pressures; and (2) these mutations do not significantly contribute to the attenuation of pathogenicity. The above results suggest that viral genome evolution is a comprehensive result of different gene changes and mutations during the process of vaccine development and that the *env* may play crucial roles in the attenuation of pathogenicity [6]. The *env* is closely related to the virus’s targeting ability and immunogenicity. Multiple lines of evidence have demonstrated that the Env of highly virulent lentiviral strains contributes to stronger neutralization escape capabilities, enterotoxic activities and the ability to activate signaling pathways of inflammatory factors compared to the Env of attenuated strains [7].

### Stable mutation rates during the development of an EIAV attenuated vaccine

**Fig. 3:**
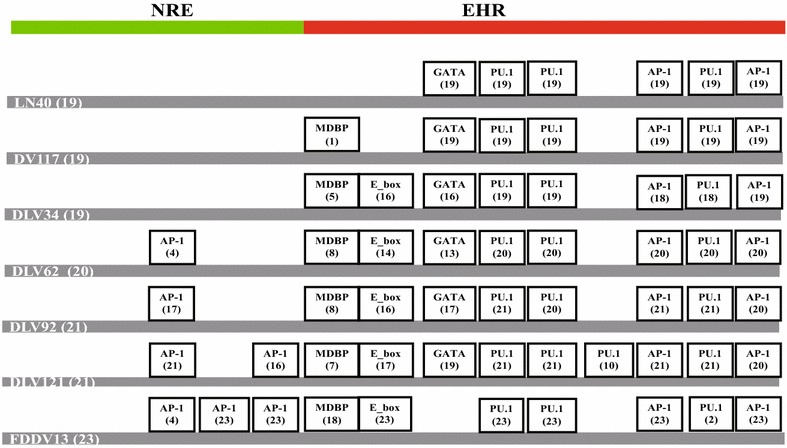
Alteration of the transcription factor binding motifs in the LTR U3 region. Sequences of 134 clones of the LTR from seven EIAV strains were randomly isolated from PCR-amplified fragments and analyzed. *NRE* indicates the negative regulation element and *EHR* indicates the enhancer region. The *boxes* define transcription factor binding sites. The numbers at the left end of the *gray lines* indicate the detected clones of each strain. The *numbers *in *parentheses* refer to the numbers of clones that contain the indicated transcription factor binding site

**Fig. 4:**
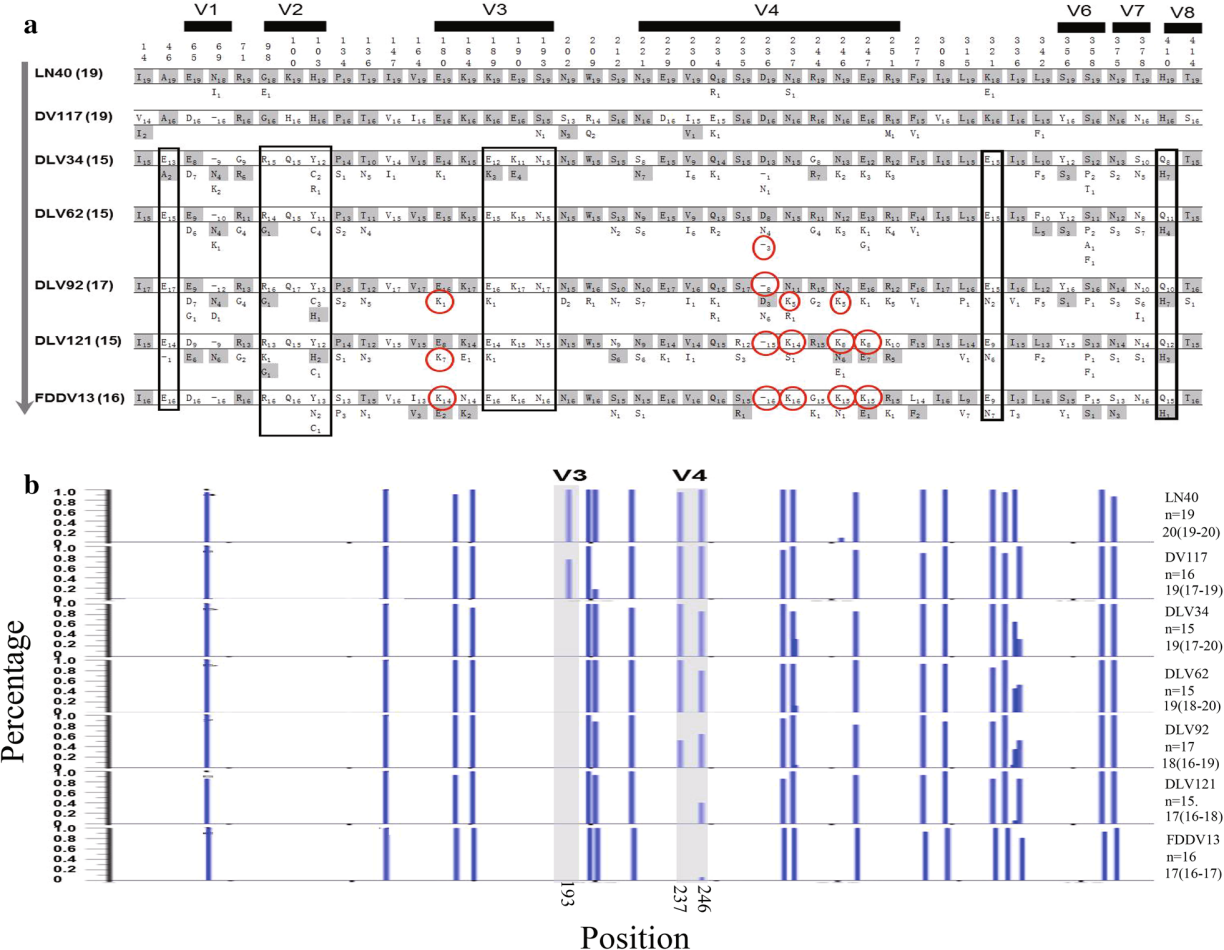
The stable mutations of EIAV gp90. **a** Stable mutations in gp90 proteins generated during different vaccine development stages. The amino acid sequences were deduced from the gene sequences originating from either the proviral genomes or from the directly cloned PCR products after removing sequences containing premature stop codons. The gp90 sequences were aligned to the reference sequence EIAV_LN40_. The *shadowed* residues and *white background* residues are identical to or different from the reference sequence, respectively. Stable mutation sites detected primarily in virus strains adapted to cultivated cells are* boxed*, whereas those limited in the attenuated strains are marked with *red circles*. V1–V8 designate the eight variable regions. The numbers on the *top of the graphs* show the positions of stable mutation sites, and those at the *left side* indicate the sequences applied for the analysis. The *downward arrows* indicate the direction of the vaccine development process. **b** The changes in gp90 glycosylation sites during vaccine development. The 116 gp90 sequences of seven different EIAV strains were analyzed using the N-GlycoSite program (http://www.hiv.lanl.gov/content/sequence/GLYCOSITE/glycosite.html). The letter “n” in the labels on the* right side* indicates the total clone number. The numbers below show the average, minimum and maximum values of predicted N-glycosylation sites. The *Y-axis* shows the percentage of each glycosylation site in the detected clones

To elucidate the meaning of the EIAV genome evolution and the contributions of the mutations in each gene to the altered viral functions during long-term in vivo and ex vivo passaging, the mutational characteristics of the LTR and viral genes were extensively analyzed using the predominant sequence of EIAV_LN40_ as the reference (Figs. 3, 4 and Additional file 2: Figure S2, Table 2). Some sequences contained premature stop codons in the coding region; these genes might play some roles in the quasispecies reservoir but were defective in the virus and were excluded from the analysis. For the convenience of description, we classified the seven viral strains according to their pathogenicity and origins into the following groups: virulent strains (EIAV_LN40_, EIAV_DV117_, EIAV_DLV34_ and EIAV_DLV62_); in vivo highly virulent strains (EIAV_LN40_ and EIAV_DV117_); attenuated strains (EIAV_DLV92_, EIAV_DLV121_ and EIAV_FDDV13_); cell culture adapted viral strains (EIAV_DLV34_, EIAV_DLV62_, EIAV_DLV92_, EIAV_DLV121_ and EIAV_FDDV13_); and vaccine strains (EIAV_DLV121_ and EIAV_FDDV13_). Some strains could be placed in more than one group because of different classification categories.

**Table 2:**
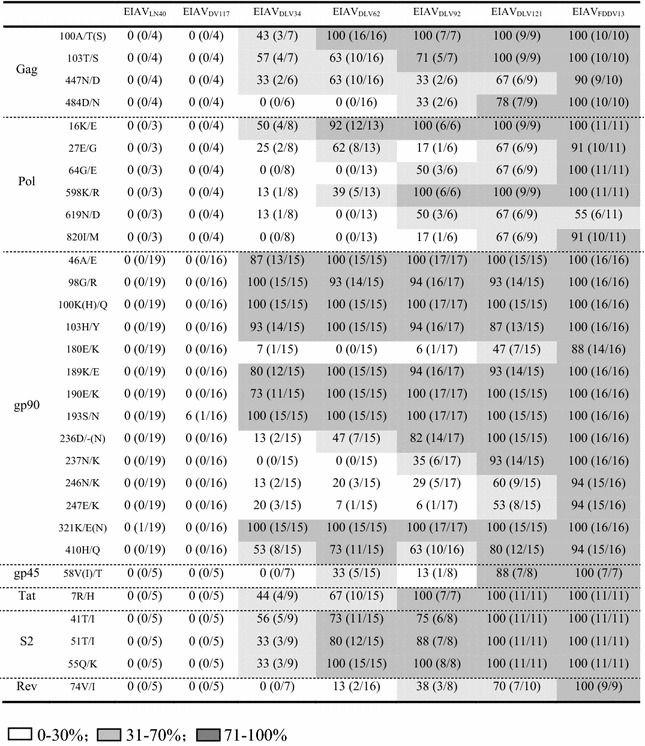
The proportion of stable amino acid substitutions of each gene in various EIAVs

The LTR region contained the highest mutation rate in the genomic sequence because of insertions, deletions and point substitutions. The mutations were largely clustered in the U3 region, which consisted of the negative regulation element (NRE) and enhancer region (EHR). Most of the deletion and insertion mutations were identified in this region and were presumed to result in changes in the number and specificity of binding sites for cellular transcription factors (Fig. 3). The E_box is the bHLH transcription factor binding sequence. It was originally absent in the two in vivo virulent strains, but it appeared in the EHR in all of the cell culture adapted virus strains. The E_box sequence in the LTR of HIV-1 and human T cell leukemia virus-1 (HTLV-1) was found to significantly decrease or delay the transcription of the provirus [8]. Additionally, sequences with AP-1 binding sites in the NRE region began to appear and gradually increased concomitantly with the successive passages in cultured cells. The clones of the most attenuated strain (EIAV_FDDV13_) contained two to three AP-1 binding sites. The transcription factor AP-1 is known to regulate cell differentiation, proliferation and apoptosis in multiple cell types. Studies of FIV revealed that the AP-1 binding sites in the U3 region improved viral replication in feline kidney cell lines but decreased viral propagation in feline T lymphocytes and PBMCs [9]. In this study, the replication ability in cultured cells of the EIAV strains was positively correlated with the AP-1 copy number and the percentage of clones with high-copy AP-1 but was negatively correlated with viral pathogenicity. The fibroblast (FDD cells)-adapted EIAV_FDDV13_ lost the GATA binding site, which was present in the EHR region in the other strains. The transcription factor GATA regulates hematopoietic differentiation and is necessary for the development, differentiation and maturation of erythrocytes [10].

A predominant mutation in coding sequences is defined as that more than 10 % of clones in different viral strains exhibit mutations causing the change of same amino acid or more than 2/3 of clones in the same viral strain display the identical mutation sites. The EIAV Gag protein is a precursor that is subsequently hydrolyzed into the p15 matrix, p26 capsid, p11 nucleocapsid and p9 proteins. Four out of 14 predominant mutations were specifically generated during the period of ex vivo attenuation (Additional file 2: Figure S2A). Among these four sites, 100A/T(S), 103T/S and 447N/D were mostly observed in the cell culture adapted viruses; the first two were located in the CTL epitope region in the E2 domain of p15 and the third was located in p9. In contrast, the 484D/N mutation was only identified in p9 in the three fully attenuated virus strains. The p9 protein is important for provirus formation and viral budding [11].

The cleavage of the EIAV Pol precursor protein generates several viral enzymes that are essential for the viral life cycle, including the reverse transcriptase (RT)/RNaseH, a viral protease, a dUTPase and an integrase. During the EIAV vaccine development process, 24 predominant mutation sites were observed in Pol (Additional file 2: Figure S2B). Among these sites, three were primarily identified in the viral strains adapted in cultured cells (16 K/E, 27E/G and 598 K/R), with the first two located in the leader sequences. Additionally, another three mutations were primarily confined to the three attenuated strains (64G/E, 619 N/D and 820I/M), with two located in the RT/RNaseH region and one in the dUTPase region. The dUTPase plays important roles in influencing pathogenicity [12]. Indeed, Pol is closely associated with viral replication and the induction of immune responses. The outcomes of these viral strain-associated mutations in EIAV Pol are less understood than the outcomes of mutations in other proteins such as Env and S2 [7, 13–15], and therefore they require further investigation.

The *env* gene was the structural gene with the highest mutation rate during vaccine development. Most of these mutations were located in the envelope surface unit gp90, which contained 43 predominant mutation sites (Fig. 4a). Impressively, nine of these mutations were confined to the cell culture adapted viral strains. Five were primarily observed in the attenuated strains (180E/K in the V3 region and 236D/-, 237N/K, 246N/K and 247E/K in the V4 region), including four that resulted in a change of an acidic amino acid to a basic amino acid. The deletion of negatively charged 236D in the V4 region may lead to a polarity change of the V4 region. Liang et al. previously demonstrated that the reverse mutation of these nine substituted residues in gp90 in the vaccine strain EIAV_FDDV13_ did significantly alter the pathogenicity of EIAV [16]. Heavy glycosylation is a common feature of lentiviral envelope proteins, and the locations and numbers of glycosylation sites are associated with viral biological characteristics [17]. We found that the EIAV strains exhibited a decrease in gp90 glycosylation sites with the increasing passages in cultured cells. The average number of glycosylation sites in the virulent strains was 19–20 compared to an average of 18 in the initially attenuated strain EIAV_DLV92_ and 17 for the vaccine strains EIAV_DLV121_ and EIAV_FDDV13_ (Fig. 4b).

The 237N/K and 246N/K substitutions in the gp90 of the attenuated strains resulted in the loss of two potential glycosylation sites (237NNTW240 and 246NETW249) in the V4 region (Fig. 4b). Additionally, all cell culture adapted viral strains lost the glycosylation site 191NSSN194 in the V3 region because of the 193S/N substitution (Fig. 4b). Han et al. reported that these substitutions reduced viral replication and sensitivity to neutralizing antibodies in cultured cells [18]. Howe et al. demonstrated that the structure of the V4 region was important for EIAV evasion of immune surveillance, and the glycosylation sites in the V4 region blocked the principle neutralizing domain (PND) in the V3 region [19]. These structural features improved the resistance to host immune responses. The EIAV V3/V4 regions and the HIV-1 V1/V2 regions are topologically similar [20]. Recently, an analysis of the HIV-1 vaccine that was assessed in the Thailand RV-144 trial suggested that antibodies targeting the V1/V2 regions of gp120, which together form a five-strand beta barrel, were correlated with immune protection [21]. Therefore, the loss of glycosylation sites in the V4 region in attenuated EIAV strains may cause viruses to expose more epitopes for immune recognition (particularly the PND in the V3 region), leading to stronger stimulation of immune responses.

Our sequencing data displayed that the diversity of gp90 a.a was the highest among other EIAV structural proteins, ranging from 1.85 ± 0.25 % for EIAV_LN40_ to 4.14 ± 0.50 % for EIAV_DV117_, which implicated a wide variation in the surface antigens in different viral clones of EIAV quasispecies. Together with constant antigen shifting, the complexity in EIAV antigen composition results in the difficulty in vaccine development. We previously reported that a proviral derivate from the vaccine strain EIAV_FDDV12_ failed to elicit immune protection like its parental strain. The reduction of gp90 variation was considered the major difference between these two types of vaccine [4].

The EIAV trans membrane protein gp45 displayed a total of 10 predominant mutations, among which 58V(I)/T was primarily detected in the vaccine strains (Additional file 2: Figure S2C). We previously demonstrated that this mutation decreased the temperature sensitivity of gp45 [22], which might affect viral infection. Furthermore, all seven analyzed EIAV_FDDV13_ genomes contained a G/A mutation at the 795th nucleotide that created a premature stop codon (^793^TGA^795^) in the *gp45* gene, resulting in a truncated gp45. The viruses expressing truncated gp45 grew significantly better in FDD cells than in horse macrophage. However, there was no significant difference in replication in horses between the two EIAV strains with different gp45 configurations [23]. The C-terminal truncated mutant may be positively selected because of its benefits for viral replication in FDD cells.

EIAV encodes three accessory proteins (Tat, S2 and Rev). Corresponding functional domains in Tat and the LTR bind to the TAR region of the EIAV genomic RNA to increase gene transcription efficiency. Our analysis revealed that Tat contained four predominant mutations, among which 7R/H was primarily found in the cell culture adapted viral strains (Additional file 2: Figure S2D). S2, which was the EIAV-encoded protein with the highest amino acid mutation rate in the vaccine strains compared to the initial EIAV_LN40_, contained six predominant mutations. Of these, 6K/R was only observed in the highly virulent EIAV_DV117_ strain, whereas 41T/I, 51T/I and 55Q/K were mostly located in the cell culture adapted adapted strains (Additional file 2: Figure S2D). We previously demonstrated that the reverse mutation of these four substituted residues in the vaccine strain EIAV_FDDV13_ compared to the residues from EIAV_DV117_ did not significantly change the replication features of this vaccine strain in cultured cells [24]. Rev assists viral RNA transportation out of the nucleus and is an important factor influencing lentivirus pathogenicity. Eleven predominant mutations were generated in Rev during the vaccine development process (Additional file 2: Figure S2D). Compared to the initial strain EIAV_LN40_, most clones of other strains displayed a deletion of residue N at position 22. Two stable mutations were principally observed in EIAV_DV117_, and a third mutation (74V/I) was primarily detected in the vaccine strains.

In most cases, 33 of the aforementioned predominant mutations were stably maintained during the attenuating process in cultured cells, but were maintained in only a portion of the clones detected from related viral strains. The percentages generally increased as the passaging in cultured cells continued and were correlated with the decrease in pathogenicity (Fig. 3 and Table 2). This evidence suggested the shifting of predominant clones in the pool of EIAV quasispecies. To evaluate the relationship between these stable mutations and the attenuation of pathogenicity and the alternation of the growth environment (from in vivo to ex vivo), a statistical analysis was performed to examine the differences between virulent and attenuated strains and between in vivo-originated and cell culture adapted strains (Table 3). With the exception of 447N/D, all other stable mutations in Gag presented in Table 3 were significantly different between the virulent and attenuated strains. Furthermore, all stable mutation sites summarized in Table 3 were significantly different between the in vivo-originated strains and the cell culture adapted strains, with the exception of a few mutations including 484D/N in Gag, 16K/E, 598K/R and 820I/M in Pol and 58V(I)/T in gp45. These data suggest that most of these stable mutations are responsible for EIAV adaptation to cultured cells and are correlated with changes in pathogenicity.

**Table 3:** Differences between various amino acid sequences or LTR sequences derived from virulent and avirulent EIAV and in vivo and cell culture derived EIAV

		Virulent^a^	Avirulent^b^	*p*	Vivo^c^	Vitro^d^	*p* ^e^
Gag	100A/T	19 (31)	25 (36)	<0.01	0 (8)	45 (49)	<0.01
	103T/S	14 (31)	24 (26)	<0.01	0 (8)	41 (49)	<0.01
	447N/D	12 (31)	16 (26)	>0.05	0 (8)	28 (49)	<0.01
	484D/N	0 (31)	17 (26)	<0.01	0 (8)	19 (49)	>0.05
Pol	16K/E	16 (28)	26 (26)	<0.01	0 (8)	19 (47)	>0.05
	27E/G	10 (28)	17 (26)	0.05 < *p* < 0.01	0 (7)	42 (47)	<0.01
	64G/E	0 (28)	20 (26)	<0.01	0 (7)	27 (47)	0.05 < *p* < 0.01
	598K/R	6 (28)	26 (26)	<0.01	0 (7)	20 (47)	>0.05
	619N/D	1 (28)	15 (26)	<0.01	0 (7)	23 (47)	0.05 < *p* < 0.01
	820I/M	0 (28)	17 (26)	<0.01	0 (7)	16 (47)	>0.05
gp90	46A/E	28 (65)	48 (48)	<0.01	0 (35)	76 (78)	<0.01
	98G/R	29 (65)	45 (48)	<0.01	0 (35)	75 (78)	<0.01
	100K/(H)Q	46 (65)	48 (48)	<0.01	16 (35)	78 (78)	<0.01
	103H/Y	30 (65)	45 (48)	<0.01	0 (35)	75 (78)	<0.01
	180E/K	1 (65)	22 (48)	<0.01	1 (35)	22 (78)	<0.01
	189K/E	27 (65)	46 (48)	<0.01	0 (35)	73 (78)	<0.01
	190E/K	27 (65)	48 (48)	<0.01	0 (35)	74 (78)	<0.01
	193S/N	31 (65)	48 (48)	<0.01	1 (35)	78 (78)	<0.01
	236D/-(N)	9 (65)	45 (48)	<0.01	0 (35)	54 (78)	<0.01
	237N/K	0 (65)	36 (48)	<0.01	0 (35)	67 (78)	<0.01
	246N/K	5 (65)	29 (48)	<0.01	0 (35)	33 (78)	<0.01
	247E/K	4 (65)	24 (48)	<0.01	0 (35)	28 (78)	<0.01
	321K/E(N)	31 (65)	48 (48)	<0.01	1 (35)	78 (78)	<0.01
	410H/Q	19 (65)	37 (48)	<0.01	0 (35)	56 (78)	<0.01
gp45	58V(I)/T	5 (32)	15 (23)	<0.01	0 (10)	26 (45)	>0.05
Tat	7R/H	14 (34)	29 (29)	<0.01	0 (10)	43 (53)	<0.01
S2	41T/I	16 (34)	28 (30)	<0.01	0 (10)	44 (54)	<0.01
	51T/I	15 (34)	28 (30)	<0.01	0 (10)	41 (54)	<0.01
	55Q/K	18 (34)	30 (30)	<0.01	0 (10)	46 (54)	<0.01
Rev	74V/I	2 (35)	19 (27)	<0.01	0 (10)	21 (52)	<0.01
LTR	AP-1	4 (77)	57 (65)	<0.01	0 (38)	61 (104)	<0.01
	MDBP	14 (77)	33 (65)	<0.01	1 (38)	46 (104)	<0.01
	E_box	30 (77)	56 (65)	<0.01	0 (38)	86 (104)	<0.01

^a^Virulent strains including EIAV_LN40_, EIAV_DV117_, EIAV_DLV34_, and EIAV_DLV62_

^b^Avirulent strains including EIAV _DLV92_, EIAV_DLV121_, and EIAV_FDDV13_

^c^In vivo strains including EIAV_LN40_ and EIAV_DV117_

^d^Ex vivo strains including EIAV_DLV34_, EIAV_DLV62_, EIAV _DLV92_, EIAV_DLV121_, and EIAV_FDDV13_

^e^Determined by the Chi square test of the SAS 9.2

## Conclusions

The vaccine development process of the attenuated EIAV vaccine is based on successive passages in different hosts and cultivated permissive cells to reduce pathogenicity while retaining immunogenicity. Viral strains collected at key stages of this process provided an important source of genomic information for studies on the molecular basis of the altered virological and immunological features. Our data demonstrated that different regions of the viral genome that are associated with decreased pathogenicity exhibited a series of stable mutations and that the percentage of mutated sites in the viral population gradually increased. These results indicated that these mutations were closely involved in pathogenicity attenuation and immunogenicity enhancement. The evolutionary analysis revealed that although the direction of evolution of the whole genome was consistent with the extent of attenuation, not all of the individual genes followed the same evolutionary pattern. This finding implicates the different contributions of each gene to the adaption of EIAV to changes in the growth environment and attenuation. Furthermore, our results suggest that there is a cumulative effect of the mutations in each individual gene on phenotypic alterations, including pathogenicity and immunogenicity. The stable mutation data presented in this article provide genetic information for future studies to determine the factors involved in viral infectivity.

## Methods

### Viruses and cells

EIAV strains were stocked in the Harbin Veterinary Research Institute, Chinese Academy of Agricultural Sciences. EIAV_LN40_ and EIAV_DV117_ were originally obtained from peripheral blood mononuclear cells (PBMCs) collected during febrile episodes from horses or donkeys infected with EIAV_LN40_ or EIAV_DV117_, respectively. The strains were stored at −80 °C prior to analysis. EIAV_DLV34_, EIAV_DLV62_, EIAV_DLV92_, EIAV_DLV121_ and EIAV_FDDV13_ were obtained as lyophilized cell culture supernatants and reconstituted in sterile cattle serum. EIAV_DLV34_, EIAV_DLV62_, EIAV_DLV92_ and EIAV_DLV121_ were rejuvenated in dMDM, and the cells were harvested when they showed cytopathic effects. EIAV_FDDV13_ was rejuvenated in FDD, and the cells were harvested 10 days after inoculation. Dynamic replication curves were performed for several passages of EIAV strains, including EIAV_DLV34_, EIAV_DLV62_, EIAV_DLV92_, EIAV_DLV121_ and EIAV_FDDV13_. The same copy number of EIAV_DLV34_, EIAV_DLV62_, EIAV_DLV92_, EIAV_DLV121_ and EIAV_FDDV13_ was used to infect cultured dMDM, which was determined by quantitative real-time revers transcription (RT)-PCR of the *gag* gene fragment [4, 6]. The supernatants were collected from the infected cells at 2, 4, 6, 8 and 10 days post-infection to measure the viral quantity by assessing the reverse transcriptase (RT) activity (Additional file 2: Figure S1) [21].

### Amplification and sequencing

The proviral DNA of EIAV_LN40_ and EIAV_DV117_ were prepared from peripheral blood mononuclear cells (PBMC) taken during febrile episodes of horses or donkeys infected by EIAV_LN40_ or EIAV_DV117_, respectively. Proviral DNA of EIAV_DLV34_, EIAV_DLV62_, EIAV_DLV92_ and EIAV_DLV121_ were obtained from cultured dMDM, EIAV_FDDV13_ proviral DNA was from FDD cells, infected by respective EIAV strains. Total cellular DNA was extracted using the OMEGA Blood DNA Kit (OMEGA, Shanghai, China) according to the manufacturer’s protocols. Proviral genomes of EIAV_LN40_, EIAV_DV117_, EIAV_DLV121_ and EIAV_FDDV13_ were cloned and sequenced from two amplified fragments using long and accurate PCR (LA-PCR) [25, 26]. The near entire proviral genome (7.9 kb of 8.2 kb) of EIAV_DLV34_, EIAV_DLV62_ and EIAV_DLV92_ proviral genomes were amplified by LA-PCR. To analyze the evolutionary characteristics of the long terminal repeat (LTR) region and the *gp90* gene in detail, specific gene fragments were also directly amplified from proviral DNA of individual virus strains and cloned individually in addition to the whole genomes. After T-A cloning of PCR products, positive clones were sent for sequencing to obtain 5–16 whole-genome sequences, 19–23 LTR sequences and 16–21 *gp90* sequences from different viral strains. All clones were obtained from indicated viral genomic fragments amplified by three to five independent PCR reactions.

### Sequence and phylogenetic analysis

Nucleotide sequences were edited and assembled using the SeqManII tool of the Lasergene DNAStar program 7.1 (DNAStar) (Madison, WI, USA, 2006). The Megalign tool (DNAStar) was used for pairwise and multiple alignments of DNA sequences and for deducing amino acid sequences. Shannon entropy was calculated using BioEdit software [27]. Diversity and distance were calculated using the Molecular Evolutionary Genetics Analysis (MEGA) program 5 and a pairwise deletion option. Phylogenetic and molecular evolutionary analyses were conducted using MEGA5 (Center for Evolutionary Functional Genomics Biodesign Institute, Arizona State University, Tempe, AZ, USA, 2011) by the neighbor joining method [28]. Bootstrap values (based on 1000 replicates) for each node were provided if they were at least 70 %. N-glycosylation sites were predicted using N-GlycoSite (http://www.hiv.lanl.gov/content/sequence/GLYCOSITE/glycosite.html). The stable mutation rates (marginal frequencies at each position of the amino acid sequence) were calculated using the MargFreq software (http://sray.med.som.jhmi.edu/SCRoftware/MargFreq/). The statistical analysis of the sequence variations was performed using SAS (Statistical Analysis System) (version 9.2, SAS Institute Inc, SAS Campus Drive, Cary, NC, USA, 2008).

### Nucleotide sequence accession numbers

Sequences generated in this study have been submitted to GenBank (accession numbers: AF327878, AF327878, HM141919, GU385362–GU385365, GU385353–GU385362, HM141909–HM141912, HM1419113–HM141918, HM1419120–HM1419123, KT806081–KT806112).

## Additional files

10.1186/s12977-016-0240-6 Protective efficacy of EIAV_DLV121_ and EIAV_FDDV13_.
10.1186/s12977-016-0240-6 Replication efficacies of EIAV strains. The growth rates of five EIAV strains (EIAVDLV34, EIAVDLV62, EIAVDLV92, EIAVDLV121 and EIAVFDDV13) in cultivated dMDM for 2, 4, 6, 8 and 10 days were compared by measuring reverse transcriptase (RT) activity. Data were collected from three independent experiments.** Figure S2.** Stable mutations in Gag (**A**), Pol (**B**), gp45 (**C**), Tat, S2 and Rev (**D**) generated during different vaccine development stages. The amino acid sequences were deduced from the gene sequences originating from either the proviral genomes or from the directly cloned PCR products after removing sequences containing premature stop codons. The sequences of each gene were aligned to the reference sequence EIAVLN40. The shadowed residues and white background residues are identical to or different from the reference sequence, respectively. Stable mutation sites detected primarily in virus strains adapted to cultivated cells are boxed, whereas those limited in the attenuated strains are marked with red circles. The numbers on the top of the graphs show the positions of stable mutation sites, and those at the left side indicate the sequences applied for the analysis. The downward arrows indicate the direction of the vaccine development process.
