# Supplementary material for: Genetic Evolution during the development of an attenuated EIAV vaccine
Source: Retrovirology. 2016 Feb 3;13:9. doi: 10.1186/s12977-016-0240-6 (PMC4738788; doi:10.1186/s12977-016-0240-6)
Supplement: Supplementary file 1 — 10.1186/s12977-016-0240-6 Protective efficacy of EIAVDLV121 and EIAVFDDV13. [file 12977_2016_240_MOESM1_ESM.doc]

Table S1 Protective efficacy of EIAV_DLV121_ and EIAV_FDDV13_

| Source | Lin and Shen^1^ | | Lin^1^ | Ma^2^ |
| --- | --- | --- | --- | --- |
| Vaccine: virulent strains | EIAV_DLV121_:EIAV_LN40_ | EIAV_DLV121_: EIAV_Wyoming_ | EIAV_DLV121_:EIAV_LN40_ | EIAV_FDDV13_:EIAV_LN40_ |
| Protective efficacy(%) | 81(25/31) | 80(8/10) | 50(2/4) | 83(5/6) |

1. Lin YZ, Shen RX, Zhu ZY, Deng XL, Cao XZ, Wang XF, Ma J, Jiang CG, Zhao LP, Lv XL, et al: **An attenuated EIAV vaccine strain induces significantly different immune responses from its pathogenic parental strain although with similar in vivo replication pattern.** *Antiviral Research* 2011, **92:**292-304.

2. Ma JA, Shi N, Jiang CG, Lin YZ, Wang XF, Wang SA, Lv XL, Zhao LP, Shao YM, Kong XG, et al: **A proviral derivative from a reference attenuated EIAV vaccine strain failed to elicit protective immunity.** *Virology* 2011, **410:**96-106.
