# Supplementary material for: Genetic Evolution during the development of an attenuated EIAV vaccine
Source: Retrovirology. 2016 Feb 3;13:9. doi: 10.1186/s12977-016-0240-6 (PMC4738788; doi:10.1186/s12977-016-0240-6)
Supplement: Supplementary file 2 — 10.1186/s12977-016-0240-6 Replication efficacies of EIAV strains. The growth rates of five EIAV strains (EIAVDLV34, EIAVDLV62, EIAVDLV92, EIAVDLV121 and EIAVFDDV13) in cultivated dMDM for 2, 4, 6, 8 and 10 days were compared by measuring reverse transcriptase (RT) activity. Data were collected from three independent experiments. Figure S2. Stable mutations in Gag (A), Pol (B), gp45 (C), Tat, S2 and Rev (D) generated during different vaccine development stages. The amino acid sequences were deduced from the gene sequences originating from either the proviral genomes or from the directly cloned PCR products after removing sequences containing premature stop codons. The sequences of each gene were aligned to the reference sequence EIAVLN40. The shadowed residues and white background residues are identical to or different from the reference sequence, respectively. Stable mutation sites detected primarily in virus strains adapted to cultivated cells are boxed, whereas those limited in the attenuated strains are marked with red circles. The numbers on the top of the graphs show the positions of stable mutation sites, and those at the left side indicate the sequences applied for the analysis. The downward arrows indicate the direction of the vaccine development process. [file 12977_2016_240_MOESM2_ESM.pdf]

FIG.S1

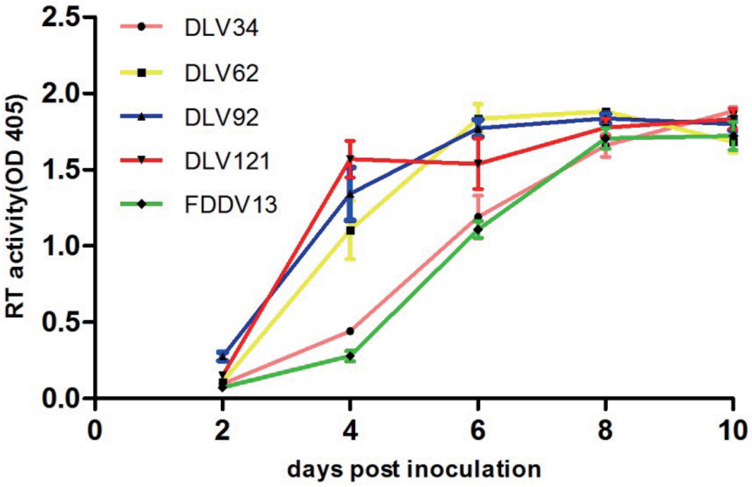

Supplementary Figure 1. Replication efficacies of EIAV strains. The growth rates of five EIAV strains (EIAV$_{DLV34}$, EIAV$_{DLV62}$, EIAV$_{DLV92}$, EIAV$_{DLV121}$ and EIAV$_{FDDV13}$) in cultivated dMDM for 2, 4, 6, 8 and 10 days were compared by measuring reverse transcriptase (RT) activity. Data were collected from three independent experiments.

FIG.S2

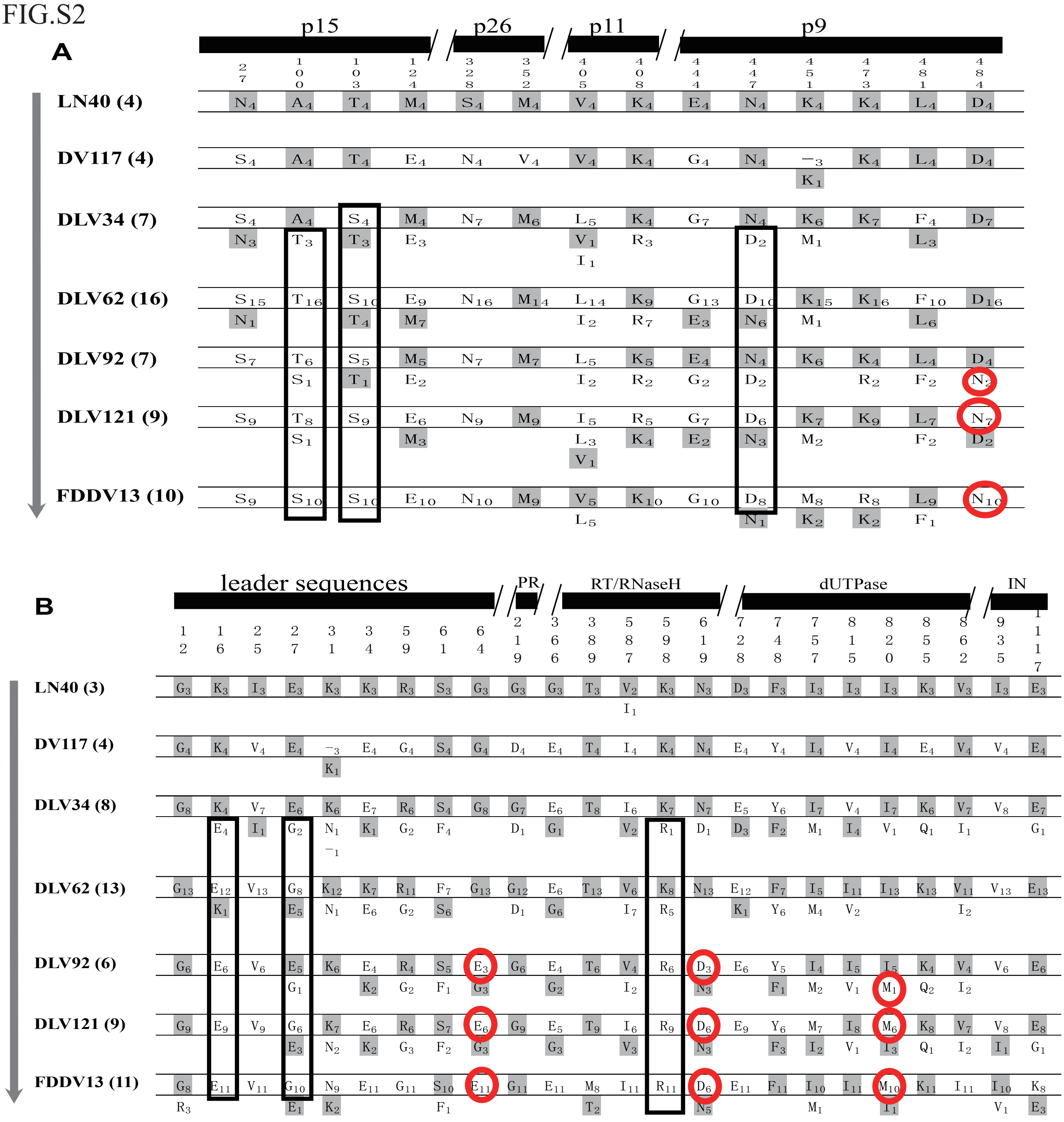

FIG.S2 continued-1

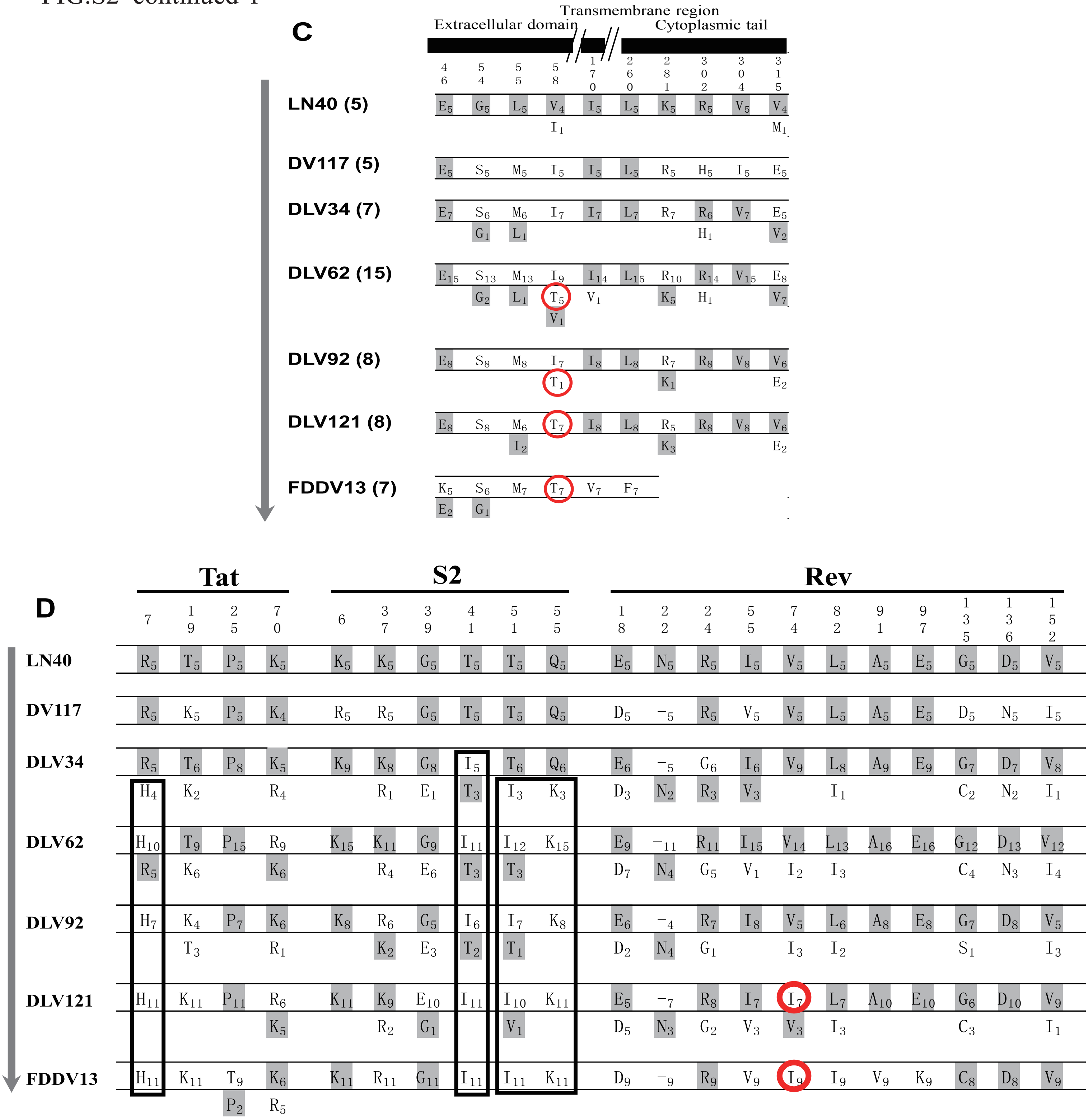

Supplementary Figure 2. Stable mutations in Gag (A), Pol (B), gp45 (C), Tat, S2 and Rev (D) generated during different vaccine development stages. The amino acid sequences were deduced from the gene sequences originating from either the proviral genomes or from the directly cloned PCR products after removing sequences containing premature stop codons. The sequences of each gene were aligned to the reference sequence $EIAV_{LN40}$. The shadowed residues and white background residues are identical to or different from the reference sequence, respectively. Stable mutation sites detected primarily in virus strains adapted to cultivated cells are boxed, whereas those limited in the attenuated strains are marked with red circles. The numbers on the top of the graphs show the positions of stable mutation sites, and those at the left side indicate the sequences applied for the analysis. The downward arrows indicate the direction of the vaccine development process.
